# Physical activity in childhood and adolescence and future depressive symptoms: an 11-year prospective cohort study

**DOI:** 10.1093/eurpub/ckad122

**Published:** 2023-08-24

**Authors:** Christopher Knowles, Kyle F Paradis, Gavin Breslin, Stephen Shannon, Angela Carlin

**Affiliations:** Sport and Exercise Sciences Research Institute, School of Sport, Ulster University, Belfast, UK; Sport and Exercise Sciences Research Institute, School of Sport, Ulster University, Belfast, UK; Bamford Centre for Mental Health and Well-being, School of Psychology, Ulster University, Coleraine, UK; Sport and Exercise Sciences Research Institute, School of Sport, Ulster University, Belfast, UK; Sport and Exercise Sciences Research Institute, School of Sport, Ulster University, Belfast, UK

## Abstract

**Background:**

Physical activity (PA) can reduce young peoples’ risk of depressive symptoms. Associations between PA and depressive symptoms are often investigated over timeframes spanning minutes to weeks. Less is known about whether childhood/adolescent PA can predict depressive symptoms in early adulthood.

**Methods:**

Using a nationally representative sample from Ireland, latent growth mixture modelling was performed to investigate the extent to which different PA trajectories existed from ages 9–17, whether gender, weight status, and socio-economic deprivation at age 9, predicted PA trajectories from ages 9–17, and whether trajectory class membership predicted depressive symptoms at age 20.

**Results:**

A 4-class solution was the best fit to the data (AIC = 52 175.69; BIC = 52 302.69; ssaBIC = 52 245.49; entropy = 1.00). Classes were labelled according to their baseline PA and slope of their trajectory: ‘High-Decreasers’; ‘Moderate-Decreasers’; ‘Moderate-Stable’; and ‘Low-Increasers’. A negative linear association existed between activity trajectory and the likelihood class members were female, overweight or socioeconomically deprived at age 9. The most active class (High-Decreasers) were significantly less likely to report depressive symptoms at age 20 than other classes.

**Conclusions:**

Multiple PA trajectories exist throughout childhood and adolescence although differences in PA levels reduced over time. The most/least active children continued to be the most/least active throughout adolescence. Those most active were least at risk of depressive symptoms in early adulthood. Being female, overweight or experiencing deprivation at age 9 were all risk factors for inactivity throughout adolescence. Findings have implications for public health and PA promotion in young people.

## Introduction

### Background

Depressive symptoms are a leading mental health-related contributor to the global burden of disease.^1^ In Ireland, 3.5% of those over the age of 15 experience chronic depressive symptoms.^2^ The peak age of onset for depressive disorders is 19.5 years with symptoms often emerging up to 5 years before diagnosis^3^ making the need for early intervention in childhood or adolescence apparent. A modifiable health behaviour consistently linked with depressive symptoms is physical activity (PA).^4^^,^^5^

In Ireland, the economic and societal effects of inactivity are substantial. It is expected that between the years 2020–30, inactivity will cause over 240 000 cases of depression.^6^ In the same period, €274 million will be spent in the provision of healthcare services managing cases of depression that may have been avoided if activity levels were sufficiently increased.^6^ To date, efforts to substantially and sustainably increase PA on a national scale have had limited success. Some evidence suggests that although PA levels tend to decrease with age,^7^^,^^8^ those most active early in life continue to be the most active in adulthood.^9^ If found to be the case in Ireland, it is possible that PA interventions targeting children may promote future activity and reduce the risk of depressive symptoms in future life stages.^10^ Moreover, the Health Behaviour in School-Aged Children 2018 survey^8^ found only 19% of girls and 29% of boys aged 13 achieve the World Health Organization and Irish Government’s recommendation of 60 min of Moderate-to-Vigorous intensity PA (MVPA) per-day inferring a large proportion of the population could benefit from increasing PA in youth.

Rates of PA participation can differ between individuals and can fluctuate over time. Studies have reported observing consistently high, low, decreasing and increasing levels of PA over time.^11^^,^^12^ This heterogeneity can obfuscate longitudinal associations between PA and mental health making inferential links between the two, indistinct. Latent growth mixture modelling (LGMM) offers a solution. LGMM facilitates the computation of multiple growth curves and assignment of participants to one of several latent trajectory classes. As such, LGMM accounts for heterogeneity that often exists between individuals’ PA levels over time, lending itself to the production of more in-depth and actionable findings than a simple unitary analysis of variance. LGMM also allows latent growth trajectories to act as both a predictor and an outcome variable. This means that not only can researchers observe the implications of class membership, they can also investigate factors likely to predict membership of high-risk classes. This information is particularly useful for effective PA promotion.

Existing research on the predictors of both PA and depressive symptoms illustrates the importance of a number of demographic and anthropometric factors. Females are more likely to be inactive than males and are more at risk for depressive symptoms.^13^^,^^14^ Whilst causality may be reciprocal, being overweight can increase risk of inactivity and depressive symptoms.^14^^,^^15^ Finally, socio-economic deprivation can increase risk of depressive symptoms and reduce involvement in mentally supportive forms of PA.^14^^,^^16^ Greater understanding of these childhood factors strengthens the ability to forecast future rates of activity and subsequent risk of depressive symptoms for different subgroups of the population.

### Study aims

The aim of the current study was to examine associations between PA performed in childhood and adolescence, and depressive symptoms in early adulthood. Specifically, we investigated the extent to which PA levels track from childhood to adolescence in a sample of Irish youth; whether gender, weight status (i.e. non-overweight, overweight), and deprivation measured in childhood were predictive of an individual’s PA trajectory throughout childhood and adolescence; and finally, whether PA trajectory throughout childhood and adolescence was predictive of depressive symptoms in early adulthood.

Existing literature has identified numerous statistically coherent models with varying numbers of PA trajectories hence, no specific predictions were made regarding the number of trajectory classes we would identify in a sample from Ireland. However, given evidence that PA levels track across the lifespan, we explored the hypothesis that the classes most/least active when aged 9 would continue to be the most/least active across all future timepoints (Hypothesis 1). We predicted that females, overweight and deprived children would all have less active PA trajectories throughout childhood and adolescence (Hypothesis 2), and that PA trajectory class membership would significantly predict depressive symptoms at age 20 with the least active class reporting the most severe symptoms (Hypothesis 3).

## Methods

The present article has been reported in line with the STrengthening the Reporting of OBservational studies in Epidemiology (STROBE) checklist.^17^

### Study design and participants

Secondary analysis was performed on data from waves 1–4 of Growing Up in Ireland (GUI): The National Longitudinal Study of Children and Young People (study methods have been published elsewhere).^18^ GUI is the first nationally representative longitudinal survey undertaken in Ireland and has collected data on four occasions beginning in 2007 when children were aged 9, with follow-up surveys at age 13, 17/18 (henceforth referred to as age 17), and 20 years old. The sample consisted of 8568 9-year-old children at baseline, 7525 (87.8%) of which were retained at age 13, 6216 (72.5%) at age 17 and 5190 (60.6%) at age 20. GUI data are freely available through the Irish Social Science Data Archive upon request. All participants surveyed at age 9 were eligible for inclusion in the current analysis (*n *=* *8568, 48.9% female), equating to ∼1 in every 7 9-year-olds (14%) resident in Ireland at the time. Written informed consent was obtained from both the child and the main caregiver prior to data collection. Ethical approval was granted by the Research Ethics Committee of the Irish Health Research Board.

### Physical activity data

A single item based on the previously validated Modifiable Activity Questionnaire was used to measure MVPA^19^. Participants were asked to report: ‘How many times in the past 14 days have you done at least 20 minutes of exercise hard enough to make you breathe heavily and make your heart beat faster?’ At age 9, wording of the item was adapted for response by the main caregiver (usually the mother). PA was recorded on a five-point scale: 1 (no days), 2 (1–2 days), 3 (3–5 days), 4 (6–8 days), 5 (9 or more days).

### Depressive symptoms

Depressive symptoms in the past week were measured at age 20 using the 8-item Centre for Epidemiological Studies Depression Scale (CES-D-8).^20^^,^^21^ The CES-D-8 has been considered a valid and reliable measure of depressive symptoms with unidimensional factor structure^21^ in young adults in Europe.^22^ For confidentiality reasons, the dataset used in the current study contained only CES-D-8 total scores rather than scores for individual items so internal consistency could not be calculated. That said, the scale has been found to have excellent internal consistency in a Czech sample of similar age (McDonalds Omega = 0.861).^22^

Depressive symptoms were rated on a scale from 0 to 24 with higher scores indicating more severe symptoms. To the best of our knowledge, a cut-off score signalling elevated symptoms has not been proposed for young adults however, a score of 9 or more has been considered an appropriate cut-off point for the screening of elevated depressive symptoms in older adults within Ireland.^23^ Exceeding the threshold for elevated symptoms should not be interpreted as sufficient for clinical diagnosis as this was not what the CES-D-8 was designed for however, consideration of this level helps provide important context surrounding symptom severity. A binary variable was derived identifying participants either having or not having elevated depressive symptoms.

### Predictors of physical activity trajectory

To offer public health authorities an insight regarding possible predictors of PA trajectories, gender, weight status and deprivation were measured at age 9. Deprivation was evaluated using the Economic and Social Research Institute (ESRI) 11-item measure of basic deprivation and consistent poverty.^24^ Those reporting the inability to afford two or more items were classed as experiencing deprivation. Body mass index (BMI) was calculated as weight in kilograms divided by height in metres squared (kg/m^2^). Gender specific International Obesity Task Force (IOTF) cut-offs for 9-year-old children were used to determine weight status whereby a BMI greater than 19.07 for boys or 18.99 for girls represented overweight.^25^

### Statistical methods

Statistical analysis was performed using Mplus version 8.8.^26^ Analysis followed the Three-Step approach to LGMM^27^ using robust maximum likelihood estimation to identify likely PA trajectories from age 9–17. The Three-Step method endorses a stepwise approach to analysis: (i) build a latent growth model without the inclusion of distal outcomes; (ii) determine likely class memberships within the sample; and (iii) analyse the association between class membership and predictors/distal outcomes. As with most national surveys, data were statistically reweighted at baseline using the 2006 Irish Census Data to ensure the sample was nationally representative of the general population.

One to six class solutions were computed to find the best fit to the data. Several model fit statistics were used to determine which solution was best: Akaike Information Criteria (AIC); Bayesian Information Criteria (BIC); sample size adjusted (ssa)BIC; and the Lo-Mendell-Rubin Adjusted Likelihood Ratio Test (LRT). For AIC, BIC and ssaBIC, lower values are representative of the best fit.^28^ LRT tests significant at *P* < 0.05 indicate the model identified is a significantly better fit to the data than a model containing one less latent class (i.e. *k* vs. *k*–1 classes). Entropy greater than or equal to 0.8 represented acceptable classification accuracy. In the event more than one model appeared to be a good fit to the data, the statistical fit was considered alongside model parsimony, with the more parsimonious solution preferred.

A series of multinomial logistic regressions were then conducted where auxiliary variables were treated as either latent class predictors or distal outcomes of trajectory class membership using the R3STEP and automatic BCH functions in Mplus, respectively.^29^^,^^30^ For predictors of latent class membership, odds ratios with 95% confidence intervals were used to interpret effect size whilst regression coefficients identified the direction of effect. Positive coefficients indicated either female, overweight or deprived children were more likely to be members of a particular class compared to the reference category. Chi-square tests were then conducted to make pairwise class comparisons of the association between class membership and distal outcomes (i.e. depressive symptoms). Auxiliary variables identified as either predictors of latent class membership or distal outcomes of latent class membership were all tested independently of one another.

## Results

In total, 8567 participants’ data were used for analysis of which 4187 (48.9%) were female, 1994 (23.3%) were experiencing deprivation, and 5750 (67.1%) were overweight at age 9. Mean (SD) PA scores for the sample were 4.17 (1.07) at age 9, 3.46 (1.22) at age 13 and 3.10 (1.32) at age 17. This equates to at least 20 min of MVPA on roughly 6, 4 and 3 days over the previous 2 weeks, respectively. At age 20, the sample reported a mean CES-D score of 4.57 (4.71). Using a cut-off score of 9, 18.4% participants reported elevated depressive symptoms at age 20. Pearson’s correlations were conducted to test relationships between all the variables used in the analysis. PA measured at age 9 (*r *=* *−0.062), age 13 (*r *=* *−0.136) and age 17 (*r *=* *−0.144) were all independently negatively correlated with depressive symptoms at age 20 at *P* < 0.01 (two-tailed).

### Modelling physical activity trajectories age 9–17

Table 1 provides an overview of the model fit statistics for 1–6 class solutions. Classification accuracy was acceptable for all models however, the 4-class model represented the most accurate solution (entropy = 1.00). LRT indicated a 4-class solution provided better fit than the 3-class solution and that 5 classes was not better than 4 classes. AIC, BIC and ssaBIC continued to decrease beyond a 4-class solution however, more complex solutions represented only marginal improvements in model fit that did not justify inclusion of additional classes. Therefore, a 4-class solution represented a quantitatively and qualitatively sound parsimonious model, and was adopted going forward.

**Table 1:** Growth mixture model fit statistics for MVPA participation from age 9–17

Classes	Loglikelihood	Best Log Replicated	AIC	BIC	ssaBIC	LRT (*P*)	Entropy
1	−34 018.249	Y	68 054.498	68 118.000	68 089.399	–	–
2	−32 500.788	Y	65 025.575	65 110.243	65 072.110	0.0000	0.945
3	−31 700.099	N	63 430.199	63 536.034	63 488.366	0.0000	0.944
4	−26 069.846	Y	52 175.691	52 302.693	52 245.493	0.0011	1.000
5	−26 052.888	Y	52 147.775	52 295.944	52 229.210	0.4804	0.832
6	−26 040.452	N	52 128.903	52 298.239	52 221.972	0.4865	0.803

**Figure 1:**
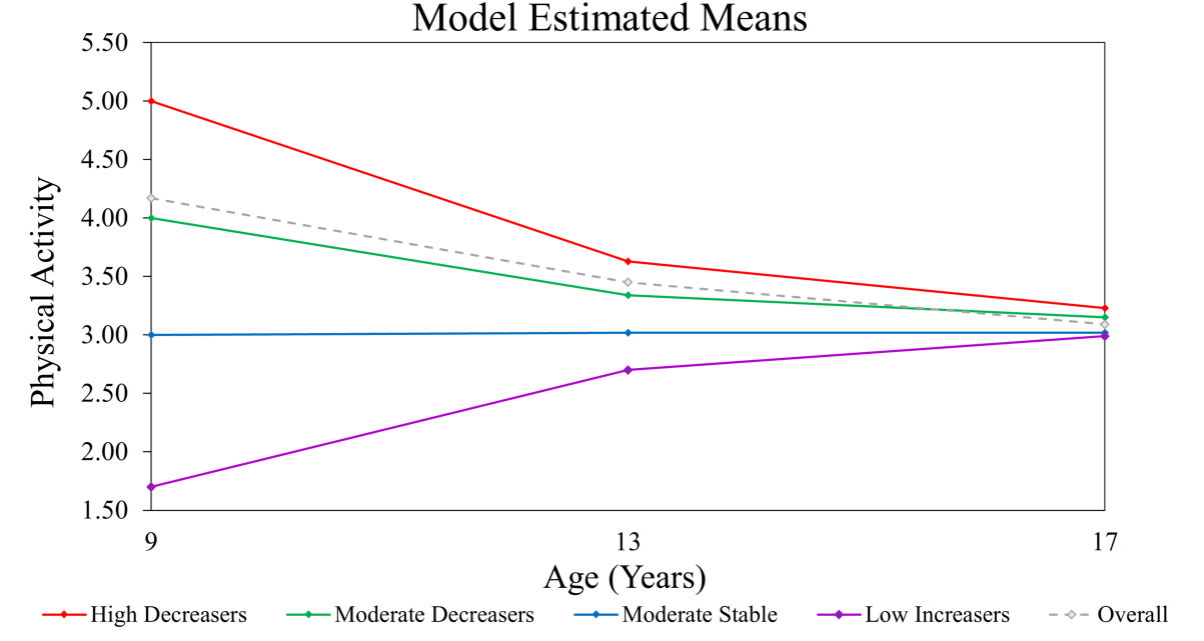
Model estimated means for physical activity trajectory throughout childhood and adolescence

Trajectories for the 4-class model are shown in figure 1. PA at age 9 which varied greatly between classes which trajectories trended towards the overall mean thereafter. Labels used to describe each class reflect first, their baseline level of activity in relation to one another (i.e. high, moderate, low) and second, the profile of their trajectory thereafter (i.e. increasing, decreasing, stable). The majority of participants (54.4%) were assigned to Class 1/High-Decreasers. Compared to other classes, High-Decreasers’ PA reduced dramatically from age 9–13 then again slightly from age 13–17. Class 2/Moderate-Decreasers (19.5%) were the second most active at age 9. Their PA levels also decreased but less dramatically than High-Decreasers. Class 3/Moderate-Stable (18.0%) reported consistent PA over time. Finally, Class 4/Low-Increasers (8.3%) were the least active at baseline but became more active over time. As the majority of the sample (54.4%) were classed as High-Decreasers, this group was used as the reference category for the next stage of analysis.

### Predictors of physical activity trajectory

Results of multinomial logistic regressions for gender, weight status and socio-economic status variables as predictors of latent class membership can be found in table 2. A linear trend existed between PA trajectories and gender, and overweight. Compared to High-Decreasers, Moderate-Decreasers, Moderate-Stable and Low-Increasers were all significantly more likely to be either female or overweight. Low-Increasers were also significantly more likely to come from a deprived background. For interpretation, less active classes were more likely to contain females and participants who were overweight at age 9 with the most inactive also more likely to have experienced socio-economic deprivation in childhood.

**Table 2:** Predictors of class membership using High-Decreasers as a reference group

	Trajectory	Standardized Beta (S.E.)	*P*	OR	95% CI	
					Lower	Upper
Gender (female)	HD	–	–	1	–	–
	MD	0.464 (0.071)	<0.001	1.590	1.384	1.828
	MS	0.526 (0.077)	<0.001	1.692	1.456	1.967
	LI	0.716 (0.110)	<0.001	2.045	1.649	2.537
Deprivation (yes)	HD	–	–	1	–	–
	MD	−0.031 (0.092)	0.740	0.970	0.809	1.162
	MS	0.081 (0.096)	0.400	1.084	0.898	1.309
	LI	0.507 (0.123)	<0.001	1.661	1.305	2.113
Weight status (overweight)	HD	–	–	1	–	–
	MD	0.248 (0.079)	0.002	1.282	1.098	1.496
	MS	0.528 (0.083)	<0.001	1.696	1.441	1.996
	LI	0.527 (0.117)	<0.001	1.694	1.346	2.132

HD, High-Decreasers; MD, Moderate-Decreasers; MS, Moderate-Stable; LI, Low-Increasers.

### Trajectory class membership predicting depressive symptoms age 20

Pairwise comparisons between adjacent classes are reported below (see table 3 for full list of comparisons). First, we tested whether participants overall depressive symptoms at age 20 differed as a function of trajectory class membership. High-Decreasers (*M *=* *4.27, *S.E*. = 0.11) reported significantly fewer symptoms than Moderate-Decreasers (*M *=* *5.02, *S.E*. = 0.19) and all other trajectory classes. No differences were identified between Moderate-Decreasers and Moderate-Stable (*M *=* *4.74, *S.E*. = 0.21), or Moderate-Stable and Low-Increasers (*M *=* *5.10, *S.E*. = 0.37). This infers only those most active throughout childhood and adolescence had a reduced risk of depressive symptoms at age 20, despite a steady decline in activity over time. Next, we tested associations between class membership and elevated depressive symptoms. High-Decreasers were significantly less likely to have elevated symptoms than Moderate-Decreasers, however, no further class differences were observed.

**Table 3:** Pairwise class comparisons of the association between PA trajectory and depression

Outcome	Class comparison	Chi-square	*d.f.*	*P*
Overall	HD vs. MD	11.586	3	0.001
Depressive	HD vs. MS	3.980	3	0.046
Symptoms	HD vs. LI	4.766	3	0.029
	MD vs. MS	.941	3	0.332
	MD vs. LI	.034	3	0.853
	MS vs. LI	.708	3	0.400
Elevated	HD vs. MD	6.291	3	0.012
Depressive	HD vs. MS	1.539	3	0.215
Symptoms	HD vs. LI	2.855	3	0.091
	MD vs. MS	.624	3	0.430
	MD vs. LI	.042	3	0.837
	MS vs. LI	.558	3	0.455

HD, High-Decreasers; MD, Moderate-Decreasers; MS, Moderate-Stable; LI, Low-Increasers.

## Discussion

This was the first study investigating the longitudinal association between PA trajectories in youth and depressive symptoms in early adulthood using a nationally representative sample from Ireland. A 4-class model was chosen as the strongest model differentiating between High-Decreasers (*n* = 4658), Moderate-Stable (*n* = 1543), Moderate-Decreasers (*n *= 1658) and Low-Increasers (*n* = 708). Although between-class differences decreased over time, relative PA levels were preserved with the most/least active in childhood remaining the most/least active throughout adolescence supporting our first hypothesis. Compared to High-Decreasers (the most active class), individuals in all other classes were more likely to be either female or overweight whilst those with the lowest activity trajectory (Low-Increasers) were also more likely to come from a deprived background supporting our second hypothesis. Compared to High Decreasers, PA trajectories from childhood through adolescence were a consistent indicator of depressive symptoms at age 20 with the least active class (Low-Increasers) reporting the most severe symptoms supporting our third hypothesis.

The trajectories modelled in the current study mirror that of existing research.^11^^,^^12^ Of the four trajectories, two groups decreased, one remained stable, and one increased over time. Interestingly, our model suggested that upon reaching 17 years of age, each classes’ activity levels were equivocal yet significant differences were observed between the depressive symptoms of the most active (High-Decreasers) and the remaining classes 3 years later. Such a finding advocates for LGMM as an informative methodological approach and highlights the importance of historic PA (specifically childhood and/or early adolescent PA) in protecting against depressive symptoms in early adulthood. Future research should investigate whether a similar relationship exists between PA trajectories and prospective depressive symptoms in older samples to ascertain whether it is specifically childhood/early adolescent PA that has a protective effect or whether prospective risk reduction occurs as a function of total PA accumulated over time at other points in the lifespan.

High-Decreasers were by far the largest class of participants and became substantially less active over time decreasing from 9 or more days of MVPA when aged 9 to roughly 3 days when aged 17. Our findings advocate for the increasingly apparent benefit of being active in childhood^31^ suggesting interventions targeting long-term increases in PA should target children early in the lifespan. However, future research should explore ways to increase (or at least reduce declining) PA rates in adolescence^32^ as this by extension, may reduce the risk of depressive symptoms further still.

When considering predictors of trajectory class membership, a linear trend emerged. Females and overweight 9-year-olds were increasingly likely to be members of less active classes. Findings suggest the prevalence of inactivity in Ireland will likely increase with rising rates of overweight in young people.^33^ Over-reliance on school-based interventions persists despite evidence that current efforts are not effective in reducing overweight nor increasing daily PA.^34^ There is need for a revised approach to intervention delivery. Past studies have found PA interventions in alternative domains (e.g. transportation, leisuretime) to be beneficial for both prospective PA levels and mental health.^35^^,^^36^ Concurrently, consideration of psychosocial factors disproportionately affecting females such as social physique anxiety^37^ is necessary given young girls’ comparatively greater risk of inactivity.

The least active class of participants were significantly more likely to come from a deprived background. We provide evidence that deprivation experienced in childhood can have a long-term impact on PA engagement which in turn increases risk of depressive symptoms over 11 years later. PA promotion should target deprived areas with consideration given to the financial implications of participation to make continued engagement in mentally supportive forms of PA (e.g. leisuretime sports) feasible for the most underprivileged and at-risk groups in society.

### Limitations

The only difference observed in prospective risk of elevated depressive symptoms was between High-Decreasers and Moderate-Decreasers implying significant mediational factors were not controlled for. Whilst the current study identified that gender, weight status, and deprivation status in childhood are predictive of PA patterns over time, it did not consider the role of additional psychosocial constructs such as those emphasized in Social Cognitive Theory^38^ and other behaviour change theories^39^ that may confound the effect of PA on depressive symptoms. Future research should consider the role of psychosocial influences such as positive self-perceptions, and social support in childhood for the formation of PA patterns over time.

Elevated depressive symptoms were reported by 18.4% of participants; a notable increase from 3.5% reporting chronic depression prior to the outbreak of the COVID-19 pandemic.^2^ Existing research suggests as with the rest of Europe, the mental health of the population in Ireland has recovered well from the pandemic.^40^ Therefore, the threshold level used to portray elevated symptoms (which is yet to be validated in young adults) should be treated with caution. Future research should investigate the extent to which scoring 9-points or above on the CES-D-8 is an appropriate level for identifying elevated symptoms in young adults.

## Conclusion

Multiple PA trajectories exist throughout childhood and adolescence and can predict risk of depressive symptoms in young adulthood. The development and execution of successful child-based PA interventions could have a marked effect on the risk of experiencing depressive symptoms in early adulthood. PA promotion should consider the role of social determinants for females and overweight children, and ease of access for those experiencing deprivation as these groups are likely to have high-risk PA trajectories. Intervention studies are required to ascertain positive means of increasing PA in childhood and sustaining that increase throughout adolescence. Statistical models should include additional theory-derived variables such as self-efficacy, and social support to strengthen understanding of factors influencing the risk of elevated symptoms. Replicating the current study in older samples would help ascertain whether the predictive effects of PA for future depressive symptoms are specific to that performed early in life or whether effects are observed throughout the lifespan.

## Data Availability

Access to data used in this study can be granted through application to the Irish Social Science Data Archive (www.ucd.ie/issda).
